# Supplementary material for: Immersive Virtual Reality Environments as Psychoanalytic Settings: A Conceptual Framework for Modeling Unconscious Processes Through IoT-Based Bioengineering Interfaces
Source: Bioengineering (Basel). 2025 Nov 17;12(11):1257. doi: 10.3390/bioengineering12111257 (PMC12650620; doi:10.3390/bioengineering12111257)
Supplement: Supplementary file 1 [file bioengineering-12-01257-s001.zip › bioengineering-3876015-supplementary.pdf]

bioengineering

MDPI

Supplementary Materials:

Figure S1. System Architecture with Behavioral Channel and I/O Flows

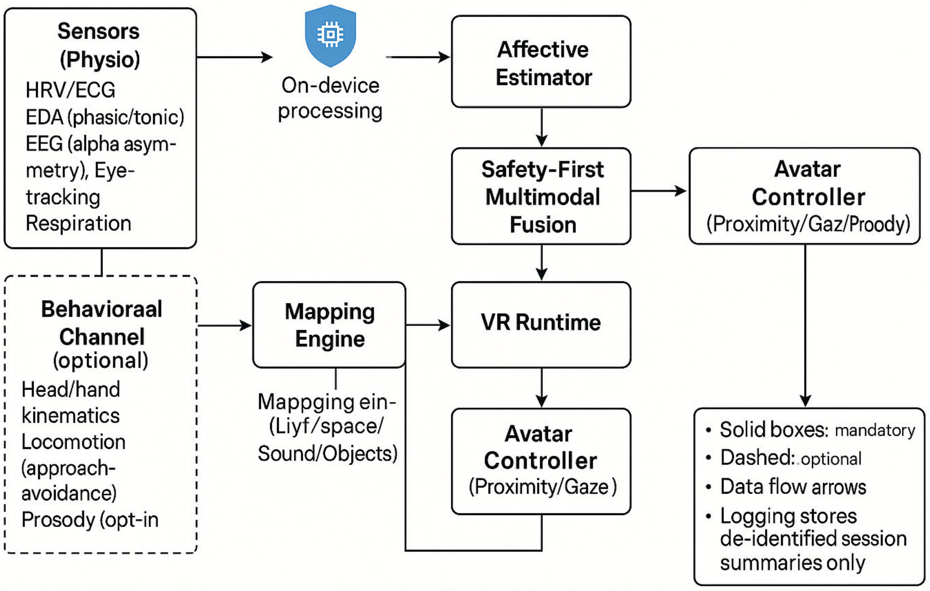

Figure S1. High-resolution technical architecture (physiological and behavioral inputs, edge pre-processing, safety-first fusion, mapping engine, VR runtime, avatar controller, de-identified logging).

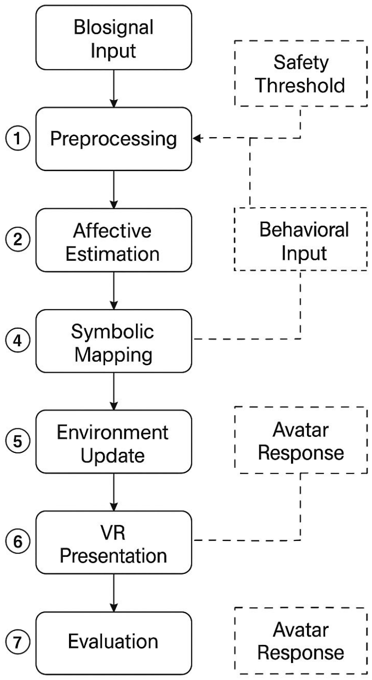

Figure S2. Numbered seven-step flow (1-7) with dashed optional modules; consistent with Figure 2 but in high

Figure S2. Numbered symbolic flow (1–7) with dashed optional modules; high-resolution version aligned to Algorithm 1.

Table S1. Extended mapping between biosignals/behavioral features and symbolic/relational parameters, including parameter ranges, trigger rules, and citations.

Signal / Feature	Physio/Behavioral Marker	Psychoanalytic Interpretation	Environmental / Avatar Modulation	Parameter Range (min/max)	Default	Trigger Rule	Reference
HRV (↓ RMSSD)	Low vagal tone; sympathetic dominance	Regression, anxiety, affective dysregulation	LightIntensity −20%; SpaceDensity +15%; AvatarProximity +0.3 m; AvatarMotion Speed −25%	RMSSD 15–60 ms	40 ms	If RMSSD z < −1 → containment mode	Shaffer & Ginsberg (2017)
EDA (↑ phasic peaks / tonic SCL)	High sympathetic arousal	Unprocessed affect; somatic projection	TransitionalObject = ON; Sound low-pass; ColorTemp 3500 K; Prosody rate −15%	SCL 1–20 µS	5 µS	If z > +1.0 → activate transitional object + reduce sound spectrum	Boucsein (2012)
EEG alpha asymmetry (R > L)	Right-lateralized alpha; withdrawal/dysphoria	Withdrawal; depressive tone	Soundscape Soothing; ColorTemp 4000 K; AvatarGaze averted; SceneContrast −10%	Alpha 8–12 Hz	10 Hz	If R>L asymmetry sustained 10s → reduce contrast, avert gaze	Allen et al. (2004)
Eye-tracking (avoidance / over-fixation; blink rate)	Gaze aversion or hyper-fixation	Resistance; hypercathexis	SymbolOpacity ±20%; PathVisibility toggle; AvatarProximity +0.2 m when avoidance>θ	Fixation 100–600 ms	300 ms	If avoidance >70% → reduce opacity; if over-fixation → introduce path toggle	Bixler & D'Mello (2015)
Respiration (shallow/irregular)	Hyperventilation; breath-holding	Repression; defensive constriction	BreathPacing 6 bpm cue; Light pulsation ±10%; SpaceDensity −10% (progressive unfolding)	RR 8–24 bpm	12 bpm	If RR >20 or <8 → cue paced breathing	Homma & Masaoka (2008)
Head/hand kinematics (variability ↓; micro-pauses ↑)	Motoric freezing / avoidance	Inhibition; fear; retreat	SpaceDensity −15%; LightIntensity −10%; AvatarProximity +0.4 m	Velocity 0.05–1.5 m/s	0.5 m/s	If velocity <0.1 m/s + micro-pauses >30 % → containment scene	Pantelopoulos & Bourbakis (2010)

Approach–avoidance distance (avatar/objects)	Relational withdrawal or engagement	Attachment dynamics; testing of safety	PathWidth ±30%; AvatarGaze on/off; AvatarProximity ±0.3 m	Distance 0.5–3 m	1.5 m	If >2.5 m → widen path; if <1.0 m → narrow path	Costantino et al. (2022)
Interaction tempo (object manipulation)	Hyper/hypo engagement	Agitation vs. apathy	SymbolicObject spawn/despawn (±50% rate); Soundscape ±6 dB; TaskHints on/off	0.1–3 Hz (actions/s)	1 Hz	If tempo <0.2 Hz → activate task hints; if >2 Hz → dampen stimuli	Gjoreski et al. (2020)

Algorithm S1. Full pseudococe de of the closed-loop controller with thresholds, safety overrides, and logging schema

function AffectiveToSymbolicControl()
  Initializize affective state zeit for vagal suppression, arousal peaks
  $\theta_{\text{vagal}} \leftarrow$ RMSSD $z < -1.0$
  θ_{arousal} – EDA peak $z + 1.0$
  loop
    loop use rate 5–10 Hz
      sense current z-HRV, EDA peaks, and EyeFix ratio values
      estimate affective state z-HRV, EDA, EyeFix ratio
      safety overrides
        if vagal suppress io detect: fallback sefalback, sefefyScene
        elseif EDA then set RegulationBlock
      compute symbolic environment: choose EyeFix [*ieg*
      set *M*appings
      wait priority rule, wait loop time or > 200 ms
    log timestamp, z-HRV, EDA peaks, EyeFix, SpaccDensity, AvatarProximity
  end

Algorithm S1. Full pseudocode of the closed-loop controller with thresholds (θ_vagal = RMSSD z < −1.0; θ_arousal = EDA peak z > +1.0), loop 5–10 Hz, safety overrides, and logging schema.
